# RETRACTION: SENP3‐Mediated TIP60 deSUMOylation is Required for DNA‐PKcs Activity and DNA Damage Repair

**DOI:** 10.1002/mco2.70954

**Published:** 2026-08-10

**Retraction: **Y. Han, X. Huang, X. Cao, Y. Li, L. Gao, J. Jia, G. Li, H. Guo, X. Liu, H. Zhao, H. Guan, P. Zhou, and S. Gao, “SENP3‐Mediated TIP60 deSUMOylation is Required for DNA‐PKcs Activity and DNA Damage Repair,” *MedComm* 3, no. 2 (2022): e123. https://doi.org/10.1002/mco2.123.

The above article, published online on 22 March 2022 in Wiley Online Library (wileyonlinelibrary.com), and its correction (https://doi.org/10.1002/mco2.197), has been retracted by agreement between the authors; the journal Editors‐in‐Chief; the Sichuan International Medical Exchange & Promotion Association (SCIMEA); and John Wiley & Sons Australia, Ltd. A third party reported on PubPeer [1] that a cropped version of the beta‐actin bands included in Figure 1E of the article and its correction statement had also been published in another article by the same author group, but representing a different detected protein [Gao et al. 2020 (https://doi.org/10.1126/sciadv.aba7822)]. The third party also reported that the IB:DNA‐PKcs (IP:ATM DNA‐PKcs) band in Figure 2C and the IB:DNA‐PKcs bands in Figure 3G were duplicated, although both figures report on different experimental conditions.

The authors responded to an inquiry by the publisher and request for raw data plus an explanation. However, the original data were no longer available.

The retraction has been agreed to because the concerns over apparent duplication of bands for separate experiments cannot be resolved without original data, and therefore the editors’ confidence in the results presented in this article has been compromised.
